# Dehydroflavonolignans from Silymarin Potentiate Transition Metal Toxicity In Vitro but Are Protective for Isolated Erythrocytes Ex Vivo

**DOI:** 10.3390/antiox10050679

**Published:** 2021-04-27

**Authors:** Zuzana Lomozová, Václav Tvrdý, Marcel Hrubša, Maria Carmen Catapano, Kateřina Macáková, David Biedermann, Radim Kučera, Vladimír Křen, Přemysl Mladěnka, Kateřina Valentová

**Affiliations:** 1 Department of Pharmacognosy, Faculty of Pharmacy in Hradec Králové, Charles University, Heyrovského 1203, 500 05 Hradec Králové, Czech Republic; lomozovz@faf.cuni.cz (Z.L.); macakovak@faf.cuni.cz (K.M.); 2 Department of Pharmacology and Toxicology, Faculty of Pharmacy in Hradec Králové, Charles University, Heyrovského 1203, 500 05 Hradec Králové, Czech Republic; tvrdyvac@faf.cuni.cz (V.T.); hrubsam@faf.cuni.cz (M.H.); 3 Department of Analytical Chemistry, Faculty of Pharmacy in Hradec Králové, Charles University, Heyrovského 1203, 500 05 Hradec Králové, Czech Republic; catapanm@faf.cuni.cz; 4 Laboratory of Biotransformation, Institute of Microbiology of the Czech Academy of Sciences, Vídeňská 1083, 142 20 Prague, Czech Republic; biedermann@biomed.cas.cz (D.B.); kren@biomed.cas.cz (V.K.); 5 Department of Pharmaceutical Chemistry and Pharmaceutical Analysis, Faculty of Pharmacy in Hradec Králové, Charles University, Heyrovského 1203, 500 05 Hradec Králové, Czech Republic; radim.kucera@faf.cuni.cz

**Keywords:** flavonolignans, silymarin, milk thistle, dehydroflavonolignans, dehydrosilybin, dehydrosilychristin, hydroxyl radical, iron, copper, prooxidation

## Abstract

2,3-Dehydrosilybin (DHS) was previously shown to chelate and reduce both copper and iron ions. In this study, similar experiments with 2,3-dehydrosilychristin (DHSCH) showed that this congener of DHS also chelates and reduces both metals. Statistical analysis pointed to some differences between both compounds: in general, DHS appeared to be a more potent iron and copper chelator, and a copper reducing agent under acidic conditions, while DHSCH was a more potent copper reducing agent under neutral conditions. In the next step, both DHS and DHSCH were tested for metal-based Fenton chemistry in vitro using HPLC with coulometric detection. Neither of these compounds were able to block the iron-based Fenton reaction and, in addition, they mostly intensified hydroxyl radical production. In the copper-based Fenton reaction, the effect of DHSCH was again prooxidant or neutral, while the effect of DHS was profoundly condition-dependent. DHS was even able to attenuate the reaction under some conditions. Interestingly, both compounds were strongly protective against the copper-triggered lysis of red blood cells, with DHSCH being more potent. The results from this study indicated that, notwithstanding the prooxidative effects of both dehydroflavonolignans, their in vivo effect could be protective.

## 1. Introduction

**Figure 1:**
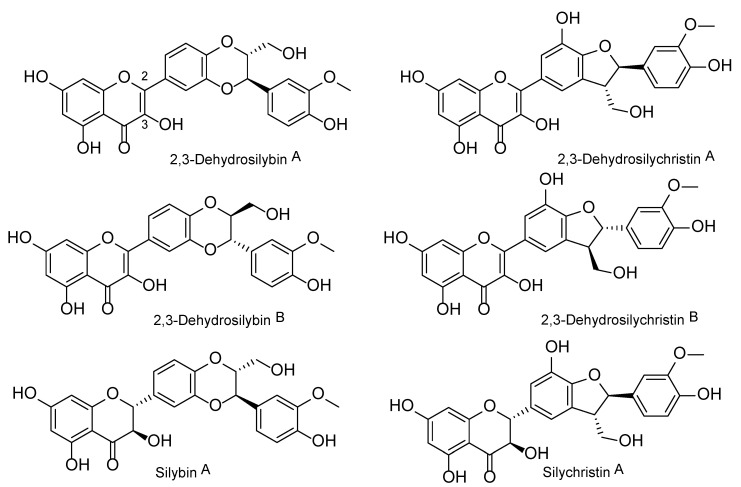
Chemical structure of 2,3-dehydrosilybin and 2,3-dehydrosilychristin enantiomers A and B, together with their respective parent flavonolignans silybin and silychristin (only A diastereomers are shown).

The 2,3-dehydroflavonolignans, such as 2,3-dehydrosilybin (DHS) and 2,3-dehydrosilychristin (DHSCH), are natural components of food supplements and other products containing silymarin—the extract from the fruits of *Silybum marianum* (L.) Gaertn. (Asteraceae) [1,2,3]. These compounds are oxidized derivatives of the major silymarin flavonolignans silybin and silychristin, and occur as mixtures of enantiomers A and B (Figure 1). Even though their occurrence was confirmed in various silymarin preparations [1,2] and they were even isolated directly from milk thistle fruits [3], it remains unclear whether they occur in intact plant material or they are artifacts formed during its processing. 2,3-Dehydroflavonolignans seem to be responsible for the distinct bioactivities of silymarin, even though they only occur in minor amounts. To date, there are only limited biological data on these silymarin constituents.

DHS, DHSCH, and 2,3-dehydrosilydianin were shown to be one to two orders of magnitude more active as direct antioxidants than the parent flavonolignans, as demonstrated by 1,1-diphenyl-2-picrylhydrazyl (DPPH) radical scavenging and microsomal lipoperoxidation assays [4,5,6,7]. Although oxygen radical absorption capacity (ORAC) of DHS and DHSCH was lower or comparable to that of silybin and silychristin, cellular antioxidant activity of DHSCH was the highest. Moreover, all compounds showed strong dose-dependent inhibition of P-gp pump and sensitization of doxorubicin-resistant ovarian carcinoma cells [8,9]. DHS was superior to silybin at inhibiting reactive oxygen species (ROS) generation in the glucose–glucose oxidase system and HepG2 cells, protection against H_2_O_2_-induced HepG2 cell death, and galactosamine-induced liver injury in vivo [10]. DHS was also found to protect against hypoxia/reperfusion injury in rat neonatal cardiomyocytes [11]. Interestingly, the same compound was also able to induce apoptosis and inhibit cancer cell invasion [10]. Further studies revealed the efficient induction of cell death in HepG2 liver and HT29 colon cancer cells by promoting their apoptosis [12] and inhibiting DNA topoisomerase I [13]. DHS also exhibited a greater ability to modulate the production of the pro-inflammatory cytokines via the NF-κB and AP-1 signaling pathways when compared to silybin and structurally related flavonoid quercetin [14]. Another interesting property of DHS is the ability to inhibit different enzymes [3,15,16]. In general, DHS, particularly DHS-B, was also more potent than silybin in complex models such as the multicellular aging model of *Caenorhabditis elegans* (lifespan extension, increased oxidative stress resistance, and inhibition of glucose transporters) [17] and mice (inhibition of basal cell carcinoma allograft tumor growth) [18]. Various semi-synthetic DHS derivatives have also been shown to possess interesting pharmacological effects [19,20,21,22,23]. Data on DHSCH are rather limited to a few studies of its antioxidant, anti-inflammatory, and multidrug resistance modulation activities [4,5,8].

Due to their structure containing the flavonoid core functionalized with hydroxy- and oxo-groups, (2,3-dehydro)flavonolignans) were suspected of interacting with transition metals and related anti/prooxidant activity. Our previous results showed that unlike silybin A, silybin B, and silychristin A, DHS was found to be a significant iron and copper chelating and copper reducing agent [24]. As far as we know, DHSCH has not been tested for such activities yet. Therefore, this study aimed to compare the chelating and reducing activities of DHS with DHSCH. In the next step, the effect of both 2,3-dehydroflavonolignans on the metal-based Fenton reaction was studied. Subsequently, in vitro Fenton reaction data were compared with ex vivo copper-triggered red blood lysis, and silymarin and its major constituents were included for comparison.

## 2. Materials and Methods

### 2.1. Chemicals

Silymarin containing silybin A (9.4%), silybin B (13.8%), silychristin A (9.5%), silychristin B (2.0%), silydianin (4.1%), isosilybin A (3.9%), isosilybin B (1.3%), isosilychristin (0.6%), taxifolin (0.8%), 2,3-dehydrosilybin (0.2%), 2,3-dehydrosilychristin (0.04%), two unknown flavonolignans (both 0.7%), and a polymeric phenolic fraction (53.0%) [2] was obtained from Liaoning Senrong Pharmaceuticals (Panjin, China, batch No. 120501). The suspension of silymarin in methanol and subsequent filtration yielded solid silybin (49.8% silybin A, 48.0% silybin B). Silychristin (87.1% silychristin A, 9.2% silychristin B) was isolated from silybin-free silymarin by LH-20 chromatography [25]. Isosilybin A (99.6%) was obtained from silybin-free silymarin by diastereomeric enzymatic resolution with Novozyme 435 and further purification [26]. DHS (95.0%) and DHSCH (97.9%) were prepared by the oxidation of silybin and silychristin, respectively [4,27].

FeSO_4_·7H_2_O, FeCl_3_·6H_2_O, CuSO_4_·5H_2_O, CuCl, hematoxylin, disodium bathocuproine disulfonate (BCS), 3-(2-pyridyl)-5,6-diphenyl-1,2,4-triazine-4’,4”-disulfonic acid sodium salt (ferrozine), hydroxylamine hydrochloride (HA), salicylic acid, disodium salt of ethylenediaminetetraacetic acid (Na_2_EDTA), hydrogen peroxide (H_2_O_2_, 30%), orthophosphoric acid (H_3_PO_4_, 85%), catechol, 2,3-dihydroxybenzoic acid, 2,5-dihydroxybenzoic acid, dithiothreitol (DTT), 2-[4-(2,4,4-trimethylpentan-2-yl)phenoxy]ethanol (Triton X), sodium acetate salt, acetic acid, 4-(2-hydroxyethyl)-1-piperazineethanesulfonic acid (HEPES), HEPES sodium salt, *tris*(hydroxymethyl)aminomethane hydrochloride (TRIS), and triethylamine (≥99.5%) were purchased from Sigma-Aldrich (Taufkirchen, Germany). Methanol (≥99.9%) and acetonitrile (≥99.9%) were from Fisher Chemical (Loughborough, UK), while HCl (35%), NaCl, and dimethylsulfoxide (DMSO) were from Penta (Chrudim, Czech Republic). Saline (NaCl, 0.9%) was from B. Braun (Melsungen, Germany). Ultrapure water (Milli-Q RG; Merck Millipore, Burlington, MA, USA) was used throughout this study.

Stock solutions of Cu/Fe salts, ferrozine, BCS (all 5 mM), and HA (100 mM) were prepared in ultrapure water except for CuCl (5 mM), which was dissolved in an aqueous solution of 0.1 M HCl and 1 M NaCl. For the HPLC method, FeCl_3_·6H_2_O (5 mM) was dissolved in the same solvent. For red blood cell lysis experiments, CuSO_4_·5H_2_O was dissolved in saline. Hematoxylin was dissolved in DMSO. Its working solution (0.25 mM) was usable for no longer than 90 min. The dehydroflavonolignans were dissolved in DMSO or in methanol (for the HPLC method).

All competitive experiments were performed in 96-well microplates (BRAND GmbH&Co. KG, Wertheim, Germany) with a Synergy HT Multi-Detection Microplate Reader spectrophotometer (BioTec Instruments, Inc., Winooski, VT, USA).

For the Fenton chemistry analysis, a chromatographic system consisting of an ESA Model 582 Solvent Delivery Module and an ESA coulometric Coulochem III detector was used (both ESA, Chelmsford, USA), equipped with a high-sensitivity analytical cell with two porous graphite working electrodes placed in series in the same cell. An Onyx Monolithic RP C8 column 100 × 4.6 mm (Phenomenex, Torrance, USA) was employed as the stationary phase. The data integration system was DataApex (Prague, Czech Republic) Chromatography Station software (version CSW 1.7).

### 2.2. pH Conditions

Experiments were performed at four pH values (4.5, 5.5, 6.8, and 7.5). Acetate buffers (15 mM of sodium acetate and 27.3 and 2.7 mM of acetic acid, respectively) were used for the two lower pH values, whereas HEPES (4-(2-hydroxyethyl)-1-piperazineethanesulfonic acid) buffers (15 mM of sodium HEPES; 71.7 and 14.3 mM of HEPES, respectively) were used for pH 6.8 and 7.5. In the Fenton chemistry experiments, TRIS buffers (5 mM) were used for pH 6.8 and 7.5 instead of HEPES buffers. The pH of these buffers was adjusted to the required value with HCl solution.

### 2.3. Iron and Copper Chelation Assessment

All the indicators used for competitive methods (ferrozine, BCS, and hematoxylin) are, in principle, also metal chelators, and they compete with the dehydroflavonolignans for metal binding. Due to the competition with the indicator, it is possible to compare the affinity of the tested flavonolignans for the metal and the stability of their complexes. First, the compounds were mixed with the metal ion to allow the complex formation, and the respective indicator was then added. The absorbance was measured immediately and again after a certain period to enable stability assessment.

An unstable complex loses its metal ion over time due to the formation of the indicator–metal complex and, hence, the absorbance at the maximum absorbance wavelength of the indicator–metal complex is significantly higher than the immediate measurement. These competitive methods are also useful for assessing reduction since both BCS and ferrozine only react with the ions in their lower valence state (Cu^+^, Fe^2+^).

#### 2.3.1. Ferrozine Method

Ferrozine is a specific reagent that forms a magenta-colored complex with Fe^2+^. Hence, this methodology is principally used to determine the chelation of ferrous ions, but it can be also used for assessing total iron (Fe^2+^ and Fe^3+^) chelation after the reduction of residual unchelated ferric ions by HA, but only at pH 4.5.

Solutions of the dehydroflavonolignans in DMSO at various concentrations were mixed for 2 min with ferrous or ferric ions (50 µL, 250 µM) in acetate (pH 4.5 and 5.5) or HEPES (pH 6.8 and 7.5) buffers. HA (50 µL, 10 mM) was added at pH 7.5 to inhibit the oxidation of ferrous ions. To assess total iron chelation at pH 4.5, the same HA aqueous solution was added after the two-minute incubation of the ferric ions with the tested compounds to reduce the remaining Fe^3+^ ions to Fe^2+^ ions. Then, ferrozine (50 µL, 5 mM) was added and the absorbance of the purple complex ferrozine-Fe^2+^ was measured immediately and after 5 min at λ = 562 nm. To determine the degree of ferric ion reduction, solutions of the dehydroflavonolignans at various concentrations were mixed for 2 min with Fe^3+^ ions in buffers. Then, ferrozine was added, and the absorbance was measured again at λ = 562 nm immediately and again after 5 min. HA was used as a positive control (100% reduction) [28].

#### 2.3.2. Hematoxylin Method

Hematoxylin forms complexes with cupric ions. DMSO solutions of dehydroflavonolignans at different concentrations were mixed with Cu^2+^ ions (50 µL, 250 µM) for 2 min in the presence of a buffer. Thereafter, hematoxylin (50 µL, 250 µM), was added. The reaction of cupric ions with hematoxylin is relatively slow and, hence, the mixture was incubated for the next 3 min to enable the formation of the complex. After incubation, the absorbance was measured immediately, and then after another 4 min (i.e., in the 7th min). Different wavelengths were used according to pH: λ = 595 nm (pH 5.5), λ = 590 nm (pH 6.8), and λ = 610 nm (pH 7.5), as previously reported [29]. Hematoxylin has a low affinity to cupric ions at pH 4.5 and, hence, the method cannot be employed at this pH.

#### 2.3.3. BCS Method

BCS is specific for cuprous ions. The methodological approaches for both reduction and chelation were almost identical to the ferrozine method, apart from the use of BCS instead of ferrozine and the employment of HA, which was added for all cuprous measurements to keep copper ions in their reduced form. The measurement was performed at λ 484 nm immediately and again after 5 min. The detailed methodology can be found in our previous study [29].

### 2.4. Effect on the Fenton Reaction Generated Hydroxyl Radicals (HPLC Method)

To monitor hydroxyl radical generation via the Fenton chemistry, a high-performance liquid chromatography (HPLC) method coupled with coulometric detection was used. The method is based on the detection and quantitation of three products which result from the reaction of the formed hydroxyl radical (^•^OH) with the indicator salicylic acid. Specifically, catechol and 2,3- and 2,5-dihydroxybenzoic acid (2,3-DHBA and 2,5-DHBA) are formed. The aim was to evaluate the antioxidant or prooxidant activity of the tested dehydroflavonolignans by comparing the formation of the hydroxylation products in the Fenton reaction mixtures with and without these compounds.

The addition of a substance with antioxidant and/or chelating properties into the reaction mixture would influence the amount of ^•^OH production, which is measured as a change in the concentrations of the three measured products. A decrease in the production of catechol, 2,3-DHBA, and 2,5-DHBA indicates a corresponding proportional decrease in ^•^OH production and is consequently related to the antioxidant activity. On the other hand, an increase in the production of the three analytes indicates that more ^•^OHs are generated and, therefore, the tested compound has prooxidant activity. It is also possible that the presence of the substance does not change the concentrations of the analytes. The methodology was recently described in detail [30]. The Fenton reaction was performed in the presence of Fe^2+^, Fe^3+^, Cu^+,^ and Cu^2+^ at four (patho)physiologically relevant pH values, as in the chelation experiments.

In the analytical cell, potential 1 was set to −200 mV and potential 2 to +450 mV, with a range of 200 nA and a +1.00 V output. The coulometric detector was also equipped with a conditioning cell set to a potential of +50 mV. The chromatograms were acquired in oxidation mode. Electrodes were cleaned at the end of each working week to prolong their use time and to obtain reproducible results. The mobile phase was a mixture of H_3_PO_4_ buffer (5 mM, pH 2.85) containing 1 mM EDTA and acetonitrile (95/5, *v*/*v*). HPLC analysis was carried out in isocratic mode at a flow rate of 1.0 mL/min and with an injection volume of 50 μL. First, 700 µL of acetate (pH 4.5 and 5.5) or TRIS (pH 6.8 and 7.5) buffer was mixed with 200 µL of methanol (blank) or solutions of dehydroflavonolignans in methanol at various concentrations. Then, 50 µL of metal ions (Fe^2+^, Fe^3+^ 1 mM and Cu^+^, Cu^2+^ 40 µM) was added so that the molar concentration ratios ranged from 200:1 to 1:50 (metal:dehydroflavonolignan). After mixing, 45 µL of salicylic acid (66.67 mM) was added. In the last step, the Fenton reaction was triggered by adding 5 µL of 30% H_2_O_2_. The reaction was carried out for 2 min at room temperature.

### 2.5. Erythrocyte Lysis Assay

This methodology has also recently been reported [31]. Blood samples were obtained from adult rats (Wistar Han, Velaz, s.r.o., Prague, Czech Republic) by exsanguination into heparinized tubes. The exsanguination was performed by a trained researcher following The Guide for the Care and Use of Laboratory Animals published by the US National Institutes of Health (8th edition, revised 2011, ISBN-13: 978-0-309-15400-0). The blood was used as a byproduct from rats after the isolation of the aorta, aimed at testing the vasodilatory effect (approval by the Czech Ministry of the Health No. MSMT-34121_2017-2).

An erythrocyte suspension was incubated with various concentrations of the flavonolignans dissolved in DMSO (maximal final concentration of DMSO was 1%) and cupric sulfate dissolved in saline at a final concentration of 500 μM for 4 h at 37 °C. The sample was then centrifuged at 1950× *g* for 10 min, and 250 μL of the supernatant obtained was used to determine the lactate dehydrogenase activity, a marker of cellular lysis. The remaining supernatant was discarded and replaced with the same amount of lysis buffer (2 mM EDTA, 1 mM DTT, 1% Triton-X 100, 0.1 M phosphate buffer of pH 7.8) as the total supernatant volume. Each sample was thoroughly vortexed and left for 20 min at room temperature to achieve complete lysis of the remaining erythrocytes. Afterward, samples were centrifuged at 6700× *g* for 10 min, and 250 μL of supernatant was used to determine lactate dehydrogenase activity.

Lactate dehydrogenase activity was kinetically quantified using the increase in absorbance caused by the conversion of β-NAD^+^ to β-NADH, employing a protocol adapted from Chan et al. [32]. Results were calculated as the percentage of erythrocytes lysed and compared to the positive control, where the solvent DMSO was used instead of the tested compound. The negative control was not treated with the metal but otherwise treated in the same way as the other samples.

### 2.6. Mathematical and Statistical Analysis

The experiments were performed with at least two independent solutions or three rat blood donor samples in at least two technical duplicates. Data are expressed as means ± SD. All statistical analyses were performed using the software GraphPad Prism version 7 for Windows (GraphPad Software, USA). For comparison of the effects of different concentrations, ANOVA with post hoc Dunnett test was used while the differences in chelation and reduction activities were analyzed by use of 95% confidence intervals of chelation curves and reduction lines.

## 3. Results

### 3.1. Copper and Iron Chelation

**Figure 2:**
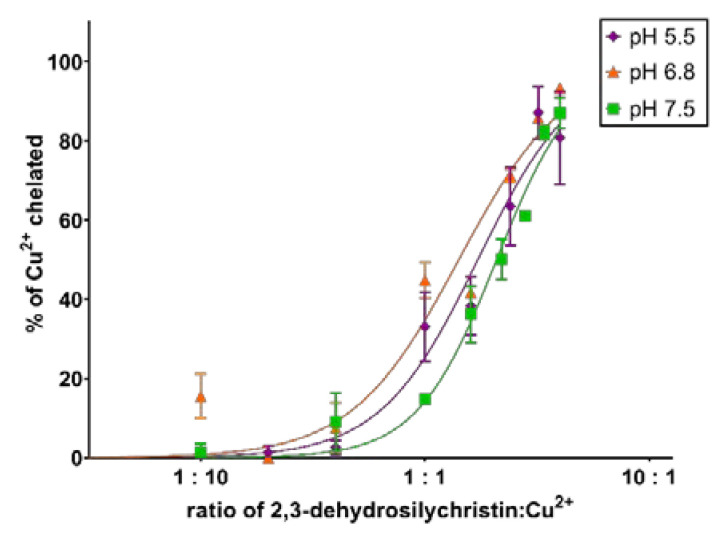
Chelation of cupric ions by 2,3-dehydrosilychristin using hematoxylin method. Data show the measurement after 5 min.

**Figure 3:**
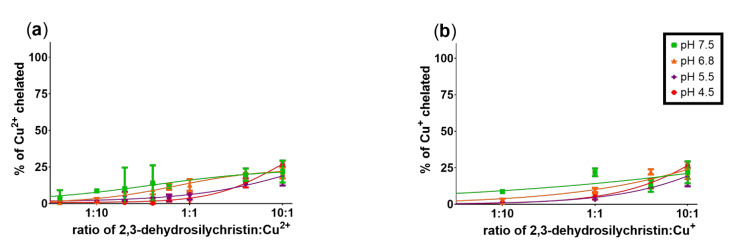
Chelation of copper ions by 2,3-dehydrosilychristin using the disodium bathocuproine disulfonate (BCS) method: (**a**) 2,3-dehydrosilychristin and cupric ions; (**b**) 2,3-dehydrosilychristin and cuprous ions. Data depict the measurement after 5 min.

In the first step, the chelation of cupric ions was screened by using the hematoxylin method. DHSCH was an active chelator that achieved complete chelation at all the tested pH levels with this method (Figure 2). The complex was highly stable at pH 5.5 and 6.8, but its stability was much lower and dependent on the concentration of the DHSCH at pH 7.5 (Supplementary Figure S1). Since this method can lead to false-positive results [33], copper chelation was further evaluated by the more precise and more competitive BCS method. The chelation activity was confirmed, but it was weak (Figure 3a). Regardless of the pH level, the chelation did not surpass 25%, even at the highest tested ratio of 10:1 DHSCH:Cu^2+^. Similar results were observed with cuprous ions (Figure 3b).

**Figure 4:**
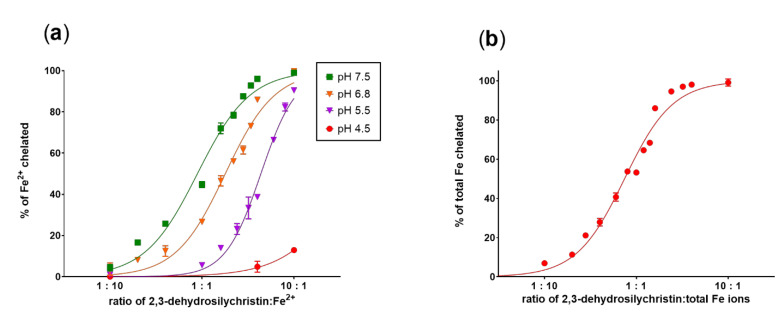
Iron-chelating properties of 2,3-dehydrosilychristin: (**a**) chelation of Fe^2+^; (**b**) total iron chelation. Data depict measurement after 5 min.

In the next step, iron chelation was also tested. At all the tested pH levels, DHSCH behaved as an iron chelator. Its ferrous ion chelating properties increased substantially with pH (Figure 4a). Total iron chelation ability was highly pronounced (Figure 4b) and the compound appeared to form a 2:1 complex with Fe^3+^ ions (e.g., at a 1:1 ratio, about 50% of the ferric ions were chelated). Similar complex formation is also likely at pH 7.5 in the case when ferrous ions were added. The complexes with ferrous ions were stable, while the stability of complexes with ferric ions was lower and clearly depended on the ratio of DHSCH to added ferric ions (Figure S2).

As we have previously reported complex data on the metal chelation of 2,3-dehydrosilybin (DHS) [24], a comparison of the metal chelation properties of both dehydroflavonolignans was performed (Figures S3–S5). The metal chelation effect was mostly comparable, but DHS was more potent under several sets of conditions. In particular, a higher copper chelating activity of DHS was observed under acidic conditions.

### 3.2. Cupric and Ferric Ion Reduction

**Figure 5:**
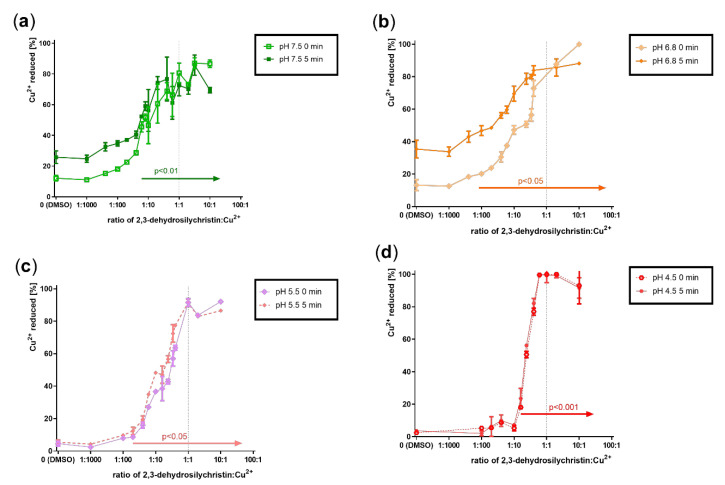
Cupric ion reduction potential of 2,3-dehydrosilychristin: (**a**) 2,3-dehydrosilychristin + Cu^2+^ at pH 7.5; (**b**) 2,3-dehydrosilychristin + Cu^2+^ at pH 6.8; (**c**) 2,3-dehydrosilychristin + Cu^2+^ at pH 5.5; (**d**) 2,3-dehydrosilychristin + Cu^2+^ at pH 4.5. The statistical comparison vs. the solvent (DMSO) is only shown for measurements performed at 5 min for a better overview.

**Figure 6:**
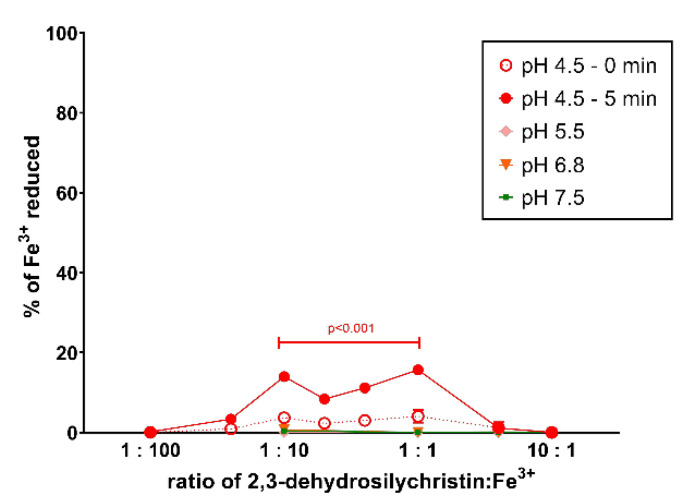
Ferric ion reduction potential of 2,3-dehydrosilychristin. Statistical comparison vs. the solvent (DMSO) is only shown for measurements performed at 5 min. No significant reduction was found at pH higher than 4.5.

As DHS and similar compounds such as flavonoids both chelate transition metals and reduce them, the cupric and ferric ion reduction property of DHSCH was also assessed. The cupric ion reduction potency of DHSCH was very high, significant from very low ratios ranging from 1:6 (pH 4.5) to 1:100 (pH 6.8) DHSCH:Cu^2+^ (Figure 5). Similarly, the reduction of ferric ions was tested. In contrast to cupric ion reduction, ferric ion reduction was only mild and observed only at pH 4.5 (Figure 6). The comparison with DHS showed that DHSCH was more potent at reducing cupric ions at pH 7.5 and 5.5, while DHS was more potent at pH 4.5 (Figure S6). With ferric ion reduction, DHSCH was slightly more potent since the maximal reduction for DHS was only around 5% [24].

### 3.3. Effect on Copper and Iron-Catalyzed Hydroxyl Radical Generation

**Figure 7:**
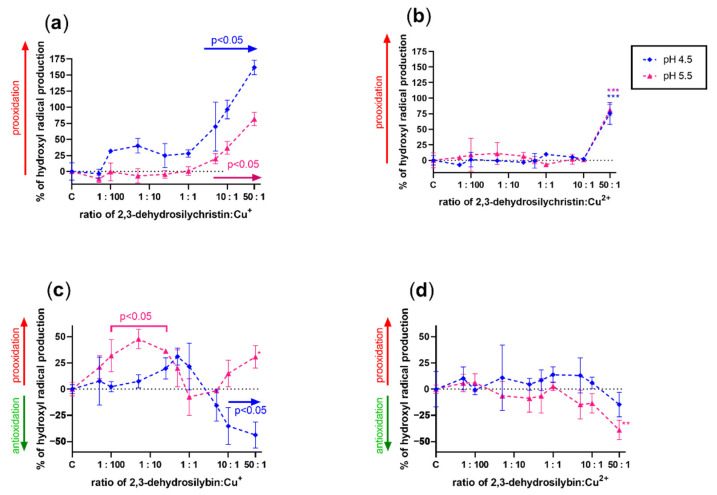
Effect of 2,3-dehydrosilychristin and 2,3-dehydrosilybin on the copper-based Fenton chemistry at pH 4.5 and 5.5: (**a**) 2,3-dehydrosilychristin + Cu^+^; (**b**) 2,3-dehydrosilychristin + Cu^2+^; (**c**) 2,3-dehydrosilybin + Cu^+^; (**d**) 2,3-dehydrosilybin + Cu^2+^. Statistical significance vs. the control (C: positive blank—the Fenton reaction with the solvent alone) is shown directly in the figure or for isolated points as * *p* < 0.05, ** *p* < 0.01, or *** *p* < 0.001.

**Figure 8:**
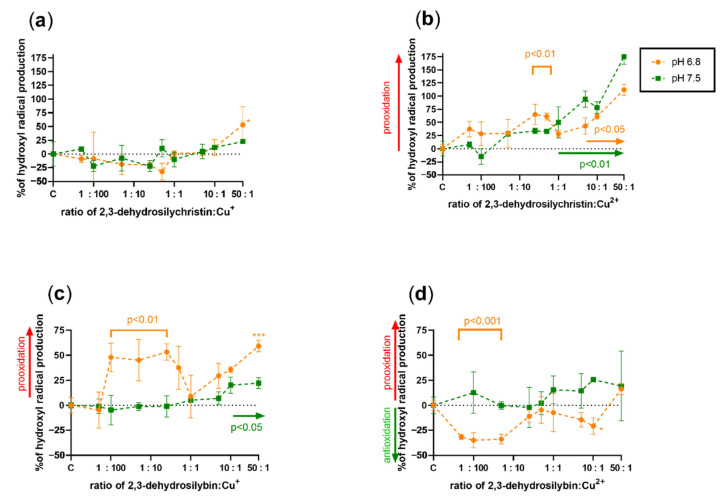
Effect of 2,3-dehydrosilychristin and 2,3-dehydrosilybin on the copper-based Fenton chemistry at pH 6.8 and 7.5: (**a**) 2,3-dehydrosilychristin + Cu^+^; (**b**) 2,3-dehydrosilychristin + Cu^2+^; (**c**) 2,3-dehydrosilybin + Cu^+^; (**d**) 2,3-dehydrosilybin + Cu^2+^. Statistical significance vs. the control (C: positive blank—the Fenton reaction with the solvent alone) is shown directly in the figure with exception of isolated points (* for *p* < 0.05 and *** *p* < 0.001).

**Figure 9:**
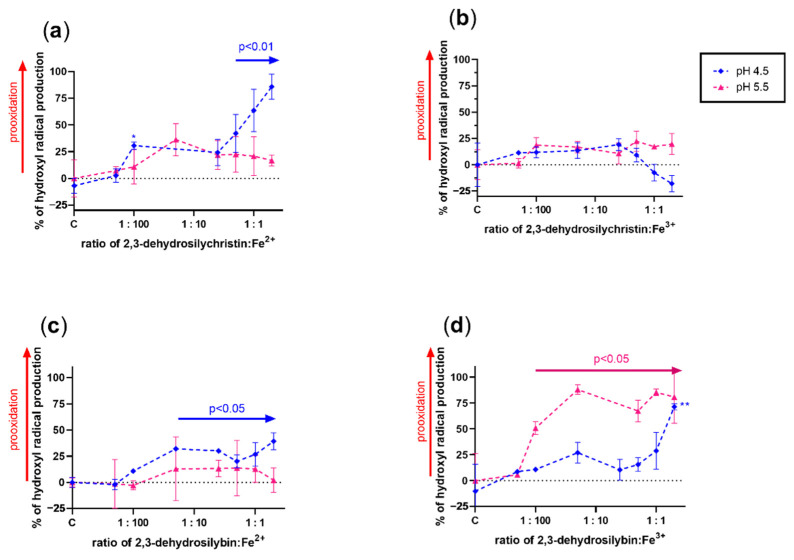
Effect of 2,3-dehydrosilychristin and 2,3-dehydrosilybin on the iron-based Fenton chemistry at pH 4.5 and 5.5: (**a**) 2,3-dehydrosilychristin + Fe^2+^; (**b**) 2,3-dehydrosilychristin + Fe^3+^; (**c**) 2,3-dehydrosilybin + Fe^2+^; (**d**) 2,3-dehydrosilybin + Fe^3+^. Statistical significance vs. the control (C: positive blank—the Fenton reaction with the solvent) is shown directly or for isolated points as * *p* < 0.05 or ** *p* < 0.01.

**Figure 10:**
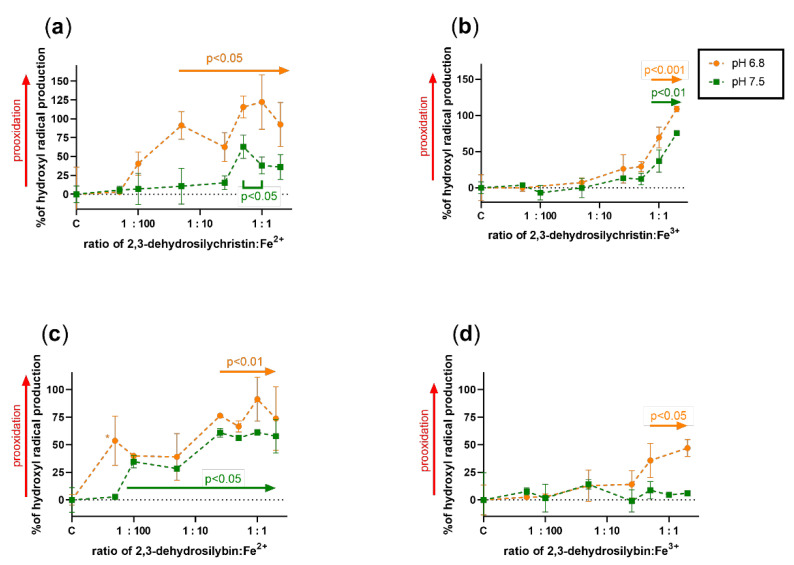
Effect of dehydroflavonolignans on the iron-based Fenton chemistry at pH 6.8 and 7.5: (**a**) 2,3-dehydrosilychristin + Fe^2+^; (**b**) 2,3-dehydrosilychristin + Fe^3+^; (**c**) 2,3-dehydrosilybin + Fe^2+^; (**d**) 2,3-dehydrosilybin + Fe^3+^. Statistical significance vs. the control (C: positive blank—the Fenton reaction with the solvent) is shown directly in the figures.

Since it is almost impossible to theoretically assess the effect of a compound that has both metal-reducing and chelating properties on Fenton chemistry, the effect of both DHSCH and DHS was tested on both copper and iron-catalyzed hydroxyl radical generation (Figure 7, Figure 8, Figure 9 and Figure 10). DHSCH always potentiated the Fenton reaction, regardless of the different oxidation states of copper or pH level. In contrast, the behavior of DHS was highly specific and often complex. At pH 4.5, it was able to attenuate the Fenton reaction in its excess over cuprous ions. It behaved similarly with cupric ions, but the effect was not significant at the same pH and became significant at pH 5.5. In contrast, DHS potentiated the Fenton chemistry driven by cuprous ions at this pH. At pH 6.8, a mild attenuation of the cupric but not cuprous-based Fenton chemistry was also observed, while at pH 7.5, only mild or neutral effects were observed for reduced or oxidized copper ions.

The effect of both dehydroflavonolignans on iron-based Fenton chemistry was always prooxidative or neutral at best. Significant inhibition was never observed in either compound, at any of the tested pH values or oxidation states. In general, but not always, DHSCH caused more prooxidation than DHS.

### 3.4. Effect on Copper-Based Oxidation of Isolated Rat Red Blood Cells

**Figure 11:**
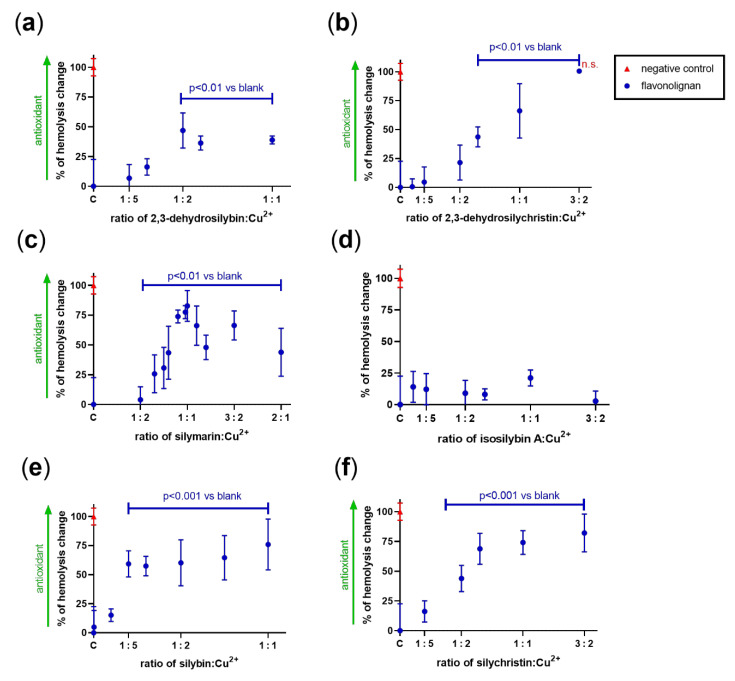
Effect of flavonolignans and silymarin on copper-triggered rat erythrocyte lysis: (**a**) 2,3-dehydrosilybin; (**b**) 2,3-dehydrosilychristin; (**c**) silymarin; (**d**) isosilybin A; (**e**) silybin; (**f**) silychristin. Only 2,3-dehydrosilychristin at the highest concentration fully abolished the hemolysis (non-significant difference vs. the negative control without copper). Some protective effect (shown in blue, significant vs. the blank, i.e., the positive control with copper) was observed in all cases except for isosilybin A. With silymarin, the molecular weight of silybin was used for calculations.

In the last step, the effect of both dehydroflavonolignans on the copper-based lysis of isolated rat red blood cells was assessed to see whether the in vitro data matched ex vivo experiments. Iron was not tested since red blood cells are generally not sensitive to iron, even at high concentrations [31]. Since direct, non-metal-related antioxidant effects can also protect erythrocytes, we also included silymarin and other flavonolignans isolated from silymarin (Figure 11), which were previously shown to be unable to chelate copper and iron [24]. Copper at a final concentration of 500 µM resulted in a profound lysis of 36.3 ± 13.5% of erythrocytes in contrast to spontaneous lysis, which was 10.9 ± 5.7%. Both dehydroflavonolignans (Figure 11a,b) were able to markedly decrease the copper-triggered lysis, but their behavior was dissimilar. The effect of DHS appeared to be plateaued, while DHSCH even reached the complete inhibition of copper-triggered hemolysis at a 3:2 ratio of DHSCH:Cu^2+^. These results do not correspond with observations from the Fenton reaction experiments. A plausible reason was direct antioxidant effects. Indeed, silymarin itself, racemic silybin, and silychristin (Figure 11c,e,f) also blocked the lysis, while isosilybin A had no effect (Figure 11d).

## 4. Discussion

Antioxidant compounds can frequently act both as antioxidants or prooxidants, depending on the conditions. The tested dehydroflavonolignans are typical examples of such anti/prooxidant compounds. Indeed, both DHS and DHSCH were documented to protect cells against oxidative stress at low concentrations, but DHS was cytotoxic to cancer or non-transformed cells at higher concentrations [5,10,12,34]. The cytostatic effect was not dependent on chirality since both DHS A and DHS B exhibited a comparable effect, suggesting a non-specific mechanism of action such as the abovementioned prooxidation [35]. Data on DHSCH available in the literature are very limited in contrast to DHS, but based on many similarities in the biological properties of both dehydroflavonolignans, an analogous biological behavior was expected. In addition, deciphering the cutoff concentration between these two effects and the related mechanism(s) is complicated by the fact that dehydroflavonolignans (1) scavenge free radicals, (2) chelate transition metals, but also (3) reduce transition metals [5,24]. It is not easy to distinguish whether their final effect will be antioxidant or prooxidant. In this study, we concentrated on their interactions with transition metals and their consequences since previous studies provide sufficient data on their scavenging effects [5,10,36]. Flavonolignans without the 2,3-double bond (silybin, isosilybin, silychristin) were not tested for chelation and in Fenton experiments in this study, since they were previously shown to only possess low chelating potential [24]. In general, they also mostly have lower scavenging effects than the corresponding dehydroflavonolignans [5,10,36]. The clear difference in the scavenging potency of flavonolignans and 2,3-dehydroflavonolignans is also maintained in their sulfated metabolites [37]. As silymarin flavonolignans are extensively metabolized in humans, mainly by phase II conjugation reactions [38], such conjugated forms are likely the biologically active forms of these compounds. Similarly to the above-reported scavenging potency, we do not expect a different chelation behavior of sulfated or glucuronidated metabolites since the sites of conjugation are mostly located far from the chelation site (e.g., position 7, 20).

**Figure 12:**
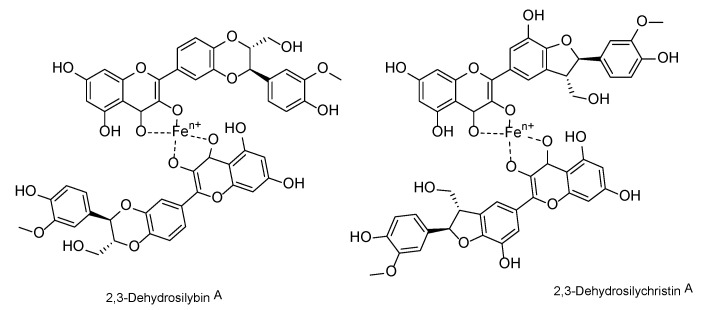
Plausible structures of the complexes of 2,3-dehydrosilybin and 2,3-dehydrosilychristin with iron.

We have previously reported on the iron and copper chelation potential of DHS [24]. This study provides fully novel data on the metal chelation of DHSCH. Although DHS and DHSCH share the same chelation site, represented by the 3-hydro-4-keto moiety (Figure 12), their chelation behavior was not identical. The difference was particularly visible for copper chelation at lower pH levels, where DHS was more potent than DHSCH. The possible interference of the chelation site with the hydroxymethyl group on the D-ring of DHSCH is the most likely explanation. The metal chelation properties of dehydroflavonolignans can, in some cases, also be responsible for the inhibition of metal-based enzymes such as collagenase and elastase [15,39]. This study also found that DHSCH was a better iron than copper chelator (Figure 3 and Figure 4). Particularly, its complexes with ferrous ions were completely stable since they retained the iron also in competition with the indicator ferrozine, which is a strong chelator (Supplementary Figure S1). Contrarily, the use of the copper indicator BCS, which is also a potent chelator, resulted in apparent removal of copper from the complex with DHSCH. This is further supported by a clear difference between mildly competitive conditions, where hematoxylin complexes with copper are formed, and highly competitive BCS with resulting low chelation potency of DHSCH (Figure 2 and Figure 3).

The metal reduction properties of both dehydroflavonolignans were indirectly previously reported by ferric reducing antioxidant power (FRAP) and Folin–Ciocãlteu reduction assays [7]. In our experiments, DHSCH was a slightly more potent ferric ion reducing agent than DHS. The latter is only a very weak reducing agent. DHSCH was also a slightly more potent cupric ion reducing agent at pH 5.5 and 7.5, but less potent at pH 4.5. Moreover, available data on the scavenging effect comparing DHS and DHSCH showed very similar activities [5,7]. The likely reason is that the same hydroxyl group in position 3 is mostly involved in the scavenging effects of both dehydroflavonolignans, in contrast to (non-dehydro)flavonolignans, where the involved hydroxyl groups are at C-20, C-19, or C-15 [5,40]. This is probably also the reason for the difference between the potency of the abovementioned antioxidant effects.

Based on these results, the theoretical assessment of the effects of these compounds on both iron- and copper-based Fenton reactions was only partly possible. This is caused by the fact that metal chelation should decrease the production of hydroxyl radicals generated by Fenton chemistry, while metal reduction will recover the catalyst (ferrous or cuprous ions) with subsequent potentiation of the reaction. Since dehydroflavonolignans are both metal chelators and reducing agents, the effect was very difficult to determine, and the performance of the experiments was necessary. The results obtained from the Fenton reaction experiments were rather complex. DHS is also both a copper reducing and chelating agent, but since its iron-chelating effect was strong and iron-reducing activity weak, an inhibition of the hydroxyl radical production was expected. However, this was not observed. The only explainable results were those with DHS and copper ions at pH 4.5 (Figure 7c,d). At lower ratios of DHS to Cu, the final effect was prooxidative or neutral because the reducing effect of DHS is initiated at low concentrations, while it was converted to a clear antioxidant effect in the presence of an excess of DHS over copper ions due to the chelating effect. The reason for the neutral or prooxidative effect of both tested dehydroflavonolignans on the iron-based Fenton reaction is not clear. It might be possible that the complex of dehydroflavonolignan with iron can still participate in Fenton chemistry, as is known for the chelator EDTA [41].

To see whether the effects on the Fenton reaction are reflected in a more complex model, the copper-based lysis of erythrocytes was selected. Iron was not included, since erythrocytes are highly resistant to even high concentrations of iron. Iron can, however, cause lipid peroxidation, and this process was previously shown to be inhibited by DHS [36]. It should be also mentioned that copper-induced lysis is a biologically relevant condition that can be relatively frequently observed in patients suffering from Wilson disease. The concentration of copper in their blood can be very high, up to 1.2 mM [42,43].

In our study, both dehydroflavonolignans protected the erythrocytes in a concentration-dependent manner, and DHSCH even achieved complete protection. To test whether chelation contributed to the effect, parent compounds with saturated C-2,C-3- bond silybin and silychristin were also included in the testing. Their effects were comparable to their corresponding dehydrogenated analogs, which suggests that copper chelation only played a marginal if any role in the process. A possible reason was the direct scavenging effect of the flavonolignans toward the reactive oxygen species formed by copper-induced hemolysis. Which reactive oxygen species are responsible for the induction of lysis is not known, but clearly the hydroxyl radical is not substantially involved, since neither of the dehydroflavonolignans were able to decrease its production in the in vitro Fenton reaction experiments. This also adds to the discussion of whether the Fenton reaction is a biologically relevant process (e.g., [44]), since the results from in vitro assays do not match with ex vivo biological experiments. Indeed, it was speculated that superoxide plays an essential role in copper-induced erythrocyte damage and, interestingly, the copper chelator 2,3-dimercaptopropane-1-sulfonate potentiated copper-based erythrocyte lysis, while most other chelators were protective [45]. Other polyphenols with both antioxidant and chelating properties were shown to also potentiate oxidative damage caused by copper in lymphocytes [46]. On the other hand, it should be emphasized that dehydroflavonolignans were more efficient cytostatics against various cancer cell lines compared to their hydrogenated counterparts, with EC_50_ in units or tens of micromolar concentrations [10,12,20,21,35]. In contrast, the cytostatic effects of DHS and silybin on fibroblasts were comparable with EC_50_ 155–160 µM [19], suggesting that the anti-/prooxidant effect can be different under pathological conditions.

## 5. Conclusions

This study reported DHSCH to be a potent iron chelator with similar efficacy to DHS under a range of pathophysiological conditions. DHSCH also chelated cupric and cuprous ions, but with a relatively low potency. Its iron-reducing potential was low, but more potent than DHS. DHSCH was, however, a strong cupric reducing agent with comparable potency to DHS. The behavior of both dehydroflavonolignans in the metal-triggered Fenton experiments showed rather prooxidative effects, which remain in clear contrast to their concentration-dependent strong protection of copper-initiated red blood cell lysis.

## Data Availability

The data presented in this study are available in the article and Supplementary Material.

## Supplementary Materials

The following are available online at https://www.mdpi.com/article/10.3390/antiox10050679/s1, Figure S1. Stability of 2,3-dehydrosilychristin complexes with cupric ions (the hematoxylin method). Figure S2. Stability of 2,3-dehydrosilychristin complexes with iron. Figure S3. Comparison of cupric-chelation activity between 2,3-dehydrosilychristin and 2,3-dehydrosilybin (hematoxylin method) with 95% confidence intervals. Figure S4. Comparison of copper-chelation activity between 2,3-dehydrosilychristin and 2,3-dehydrosilybin (the BCS method) with 95% confidence intervals. Figure S5. Comparison of iron chelating activity between 2,3-dehydrosilychristin and 2,3-dehydrosilybin with 95% confidence intervals. Figure S6. Comparison of cupric reduction between 2,3-dehydrosilychristin and 2,3-dehydrosilybin using 95% confidence intervals of linear regression lines.

## Abbreviations

^•^OH	hydroxyl radicals
2,3-DHBA	2,3-dihydroxybenzoic acid
2,5-DHBA	2,5-dihydroxybenzoic acid
β-NAD	β-nicotinamide adenine dinucleotide
BCS	disodium bathocuproine disulfonate
DHS	2,3-dehydrosilybin
DHSCH	2,3-dehydrosilychristin
DMSO	dimethylsulfoxide
DPPH	1,1-diphenyl-2-picrylhydrazyl
DTT	dithiothreitol
EDTA	ethylenediaminetetraacetic acid
FRAP	ferric reducing antioxidant power
HA	hydroxylamine hydrochloride
HEPES	4-(2-hydroxyethyl)-1-piperazineethanesulfonic acid
HPLC	high-performance liquid chromatography
ROS	reactive oxygen species
TRIS	*tris*(hydroxymethyl)aminomethane
