# Supplementary material for: Dehydroflavonolignans from Silymarin Potentiate Transition Metal Toxicity In Vitro but Are Protective for Isolated Erythrocytes Ex Vivo
Source: Antioxidants (Basel). 2021 Apr 27;10(5):679. doi: 10.3390/antiox10050679 (PMC8146032; doi:10.3390/antiox10050679)
Supplement: Supplementary file 1 [file antioxidants-10-00679-s001.zip › antioxidants-1164660-supplementary.pdf]

Dehydroflavonolignans from Silymarin Potentiate Transition Metal Toxicity in Vitro But Are Protective on Isolated Erythrocytes ex Vivo

Zuzana Lomozová[&1], Václav Tvrdý[&2], Marcel Hrubša[2], Maria Carmen Catapano[3], Kateřina Macáková[1], David Biedermann[4], Radim Kučera[5], Vladimír Křen[4], Přemysl Mladěnka[2,*] & Kateřina Valentová[4,*]

[1] Department of Pharmacognosy, Faculty of Pharmacy in Hradec Králové, Charles University, Heyrovského 1203, 500 05 Hradec Králové, Czech Republic
[2] Department of Pharmacology and Toxicology, Faculty of Pharmacy in Hradec Králové, Charles University, Heyrovského 1203, 500 05 Hradec Králové, Czech Republic
[3] Department of Analytical Chemistry, Faculty of Pharmacy in Hradec Králové, Charles University, Heyrovského 1203, 500 05 Hradec Králové, Czech Republic
[4] Laboratory of Biotransformation, Institute of Microbiology of the Czech Academy of Sciences, Vídeňská 1083, 142 20 Prague, Czech Republic
[5] Department of Pharmaceutical Chemistry and Pharmaceutical Analysis, Faculty of Pharmacy in Hradec Králové, Charles University, Heyrovského 1203, 500 05 Hradec Králové, Czech Republic

Table of Contents

Figure S1. Stability of 2,3-dehydrosilychristin complexes with cupric ions (the hematoxylin method)....................2

Figure S2. Stability of 2,3-dehydrosilychristin complexes with iron. 2

Figure S3. Comparison of cupric-chelation activity between 2,3-dehydrosilychristin and 2,3-dehydrosilybin (hematoxylin method) with 95% confidence intervals. 3

Figure S4. Comparison of copper-chelation activity between 2,3-dehydrosilychristin and 2,3-dehydrosilybin (the BCS method) with 95% confidence intervals. 4

Figure S5. Comparison of iron chelating activity between 2,3-dehydrosilychristin and 2,3-dehydrosilybin with 95% confidence intervals....................5

Figure S6. Comparison of cupric reduction between 2,3-dehydrosilychristin and 2,3-dehydrosilybin using 95% confidence intervals of linear regression lines. 6

References....................6

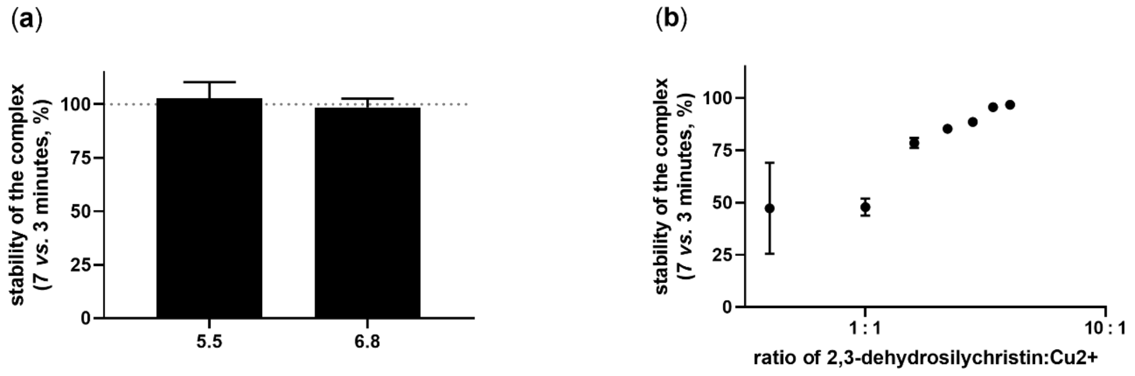

Figure S1. Stability of 2,3-dehydrosilychristin complexes with cupric ions (the hematoxylin method).

(**a**): at pH 5.5 and 6.8, (**b**): at pH 7.5. At lower pH, the complexes were clearly stable but at neutral pH, the complexes were in lower ratios unstable and the stability was apparently dependent on the ratio of 2,3-dehydrosilychristin to cupric ions. Calculations were performed as the difference between the percentage of copper chelated at 7 minutes *vs.* initial measurement.

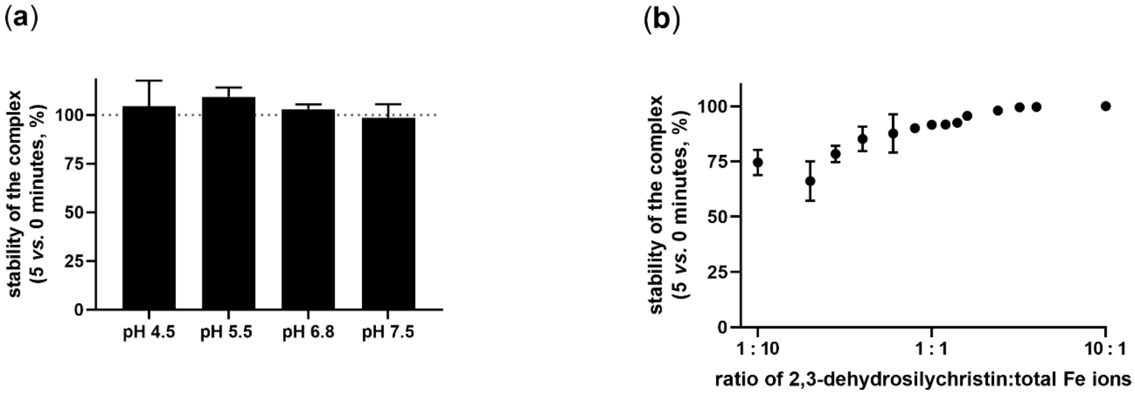

Figure S2. Stability of 2,3-dehydrosilychristin complexes with iron.

(**a**): ferrous ions, (**b**): ferric ions. While complexes with ferrous ions were very stable, the stability of the complexes with ferric ions was dependent on the ratio of 2,3-dehydrosilychristin to added ferric ions. Calculations were performed as the difference between % of copper chelated at 5 minutes *vs.* initial measurement.

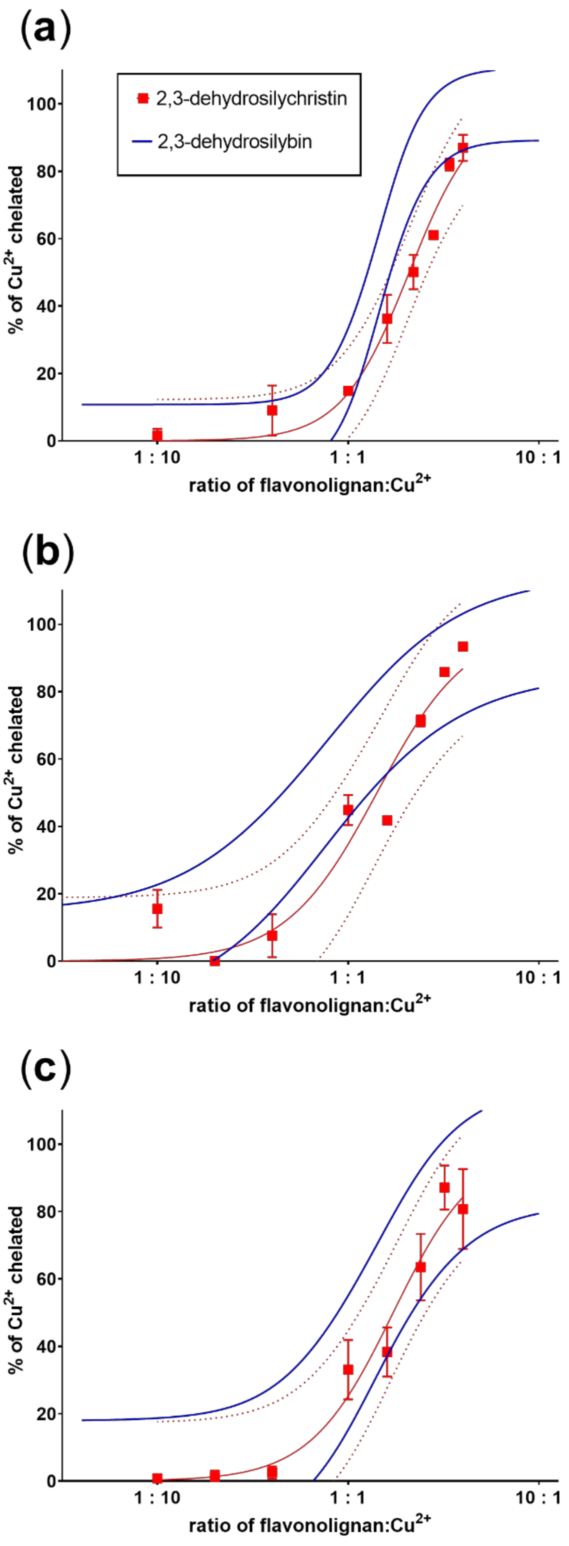

Figure S3. Comparison of cupric-chelation activity between 2,3-dehydrosilychristin and 2,3-dehydrosilybin (hematoxylin method) with 95% confidence intervals.

(**a**): pH 7.5, (**b**): pH 6.8 and (**c**): pH 5.5. At both lower pH values, there were no significant differences between chelation potency of both dehydroflavonolignans while at pH 7.5, dehydrosilybin was apparently slightly but significantly more potent. Data for dehydrosilybin were taken from the article of Tvrdý et al., 2018 and are shown as confidence intervals only for better comprehensibility.

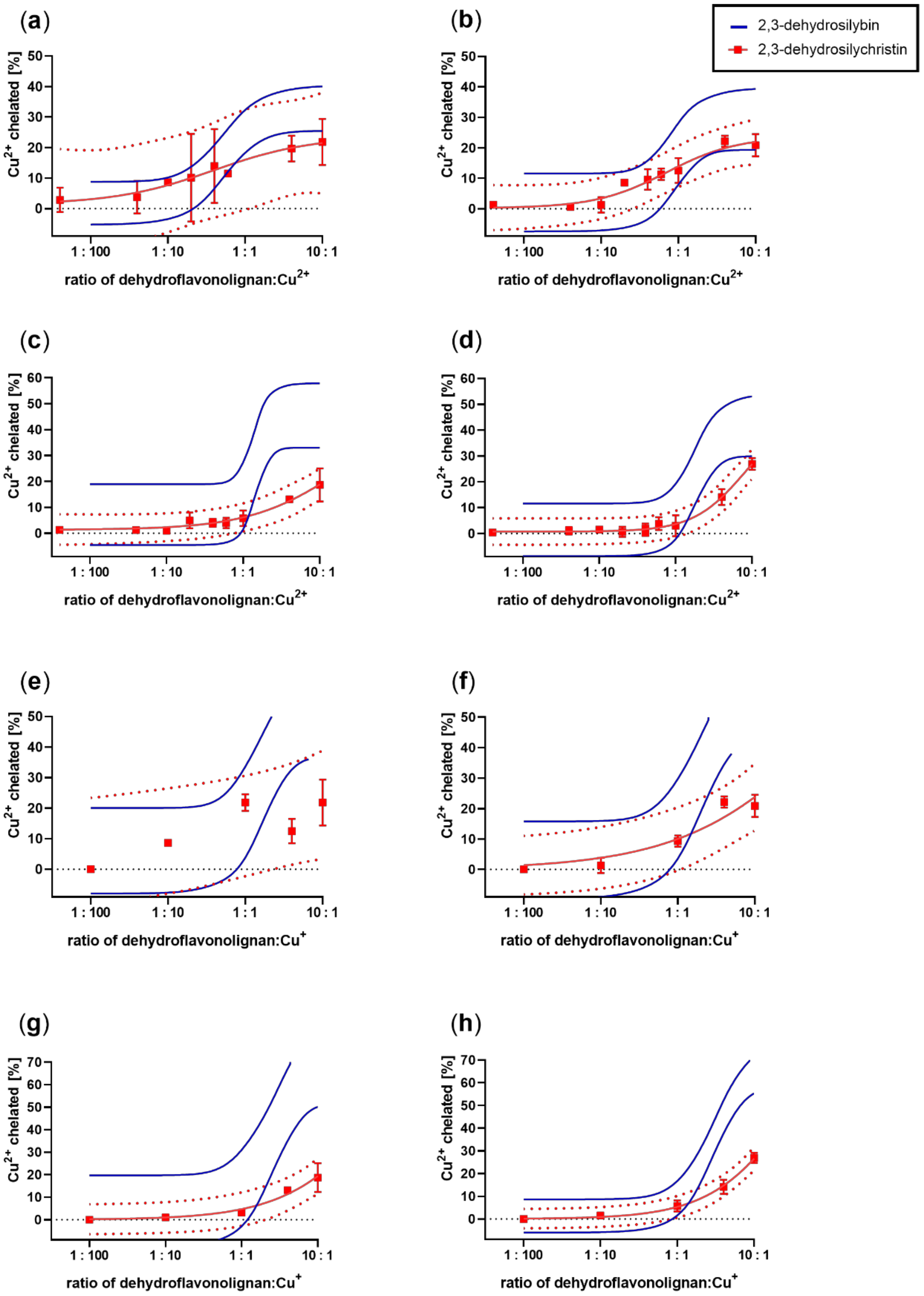

Figure S4. Comparison of copper-chelation activity between 2,3-dehydrosilychristin and 2,3-dehydrosilybin (the BCS method) with 95% confidence intervals.

(**a**)-(**d**): cupric ions, (**e**)-(**h**): cuprous ions, (**a**) and (**e**): pH 7.5, (**b**) and (**f**): pH 6.8, (**c**) and (**g**): pH 5.5, (**d**) and (**h**): pH 4.5. With exception of pH 7.5 and cupric ions at pH 6.8, 2,3-dehydrosilybin was more potent than 2,3-dehydrosilychristin. Data for dehydrosilybin were taken from the article Tvrdý et al., 2018 – only confidence intervals are shown for better comprehensibility.

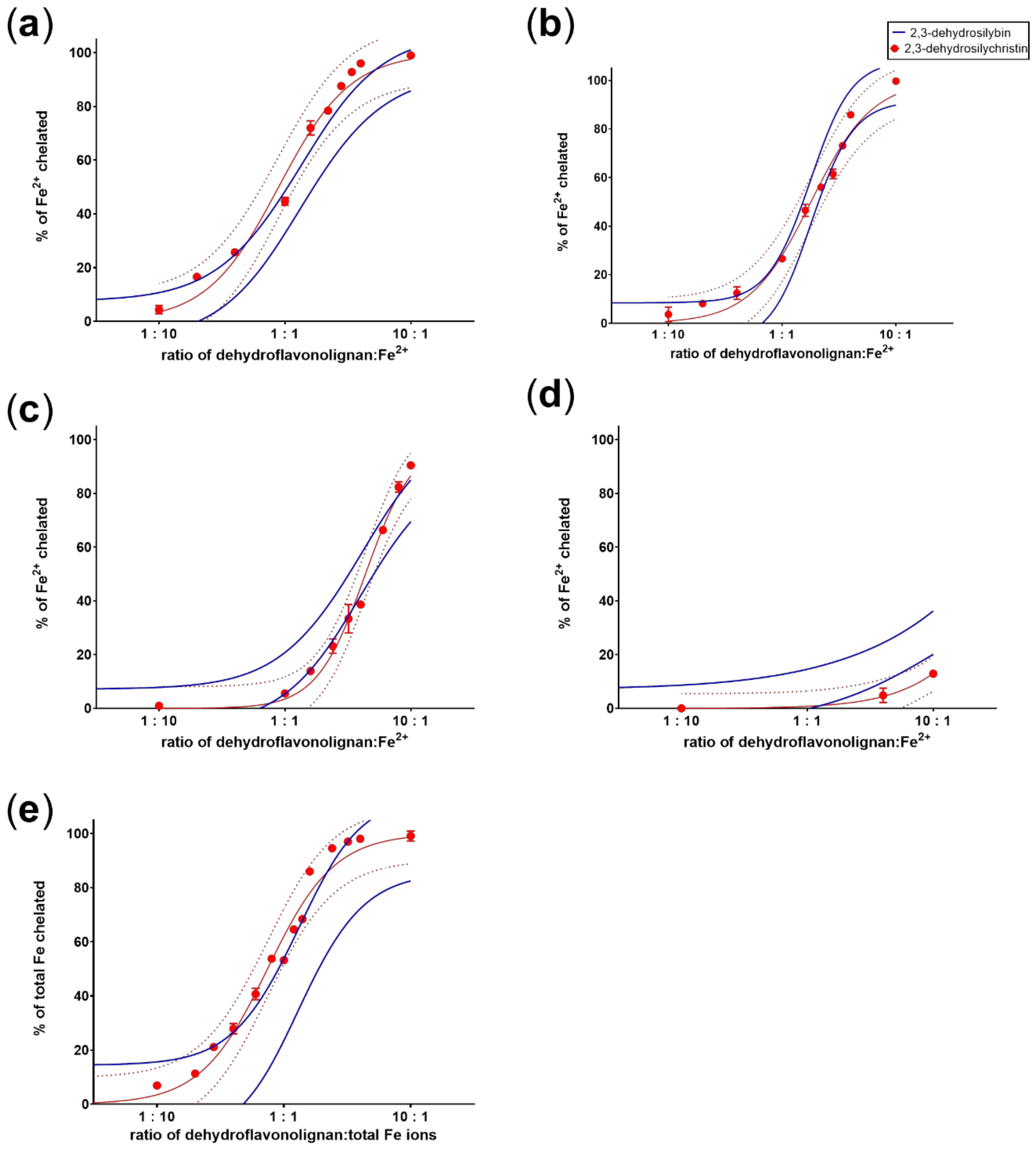

Figure S5. Comparison of iron chelating activity between 2,3-dehydrosilychristin and 2,3-dehydrosilybin with 95% confidence intervals.

(**a**): pH 7.5 and ferrous ions, (**b**): pH 6.8 and ferrous ions (**c**): pH 5.5 and ferrous ions, (**d**): pH 4.5 and ferrous ions, (**e**): pH 4.5 and ferric ions. With exception of pH 4.5 and ferrous ions (part **d**), where 2,3-dehydrosilybin was slightly but significantly more potent than 2,3-dehydrosilychristin, there were no differences between both dehydroflavonolignans. Data for dehydrosilybin were taken from the article Tvrdý et al., 2018 – only confidence intervals are shown for better comprehensibility.

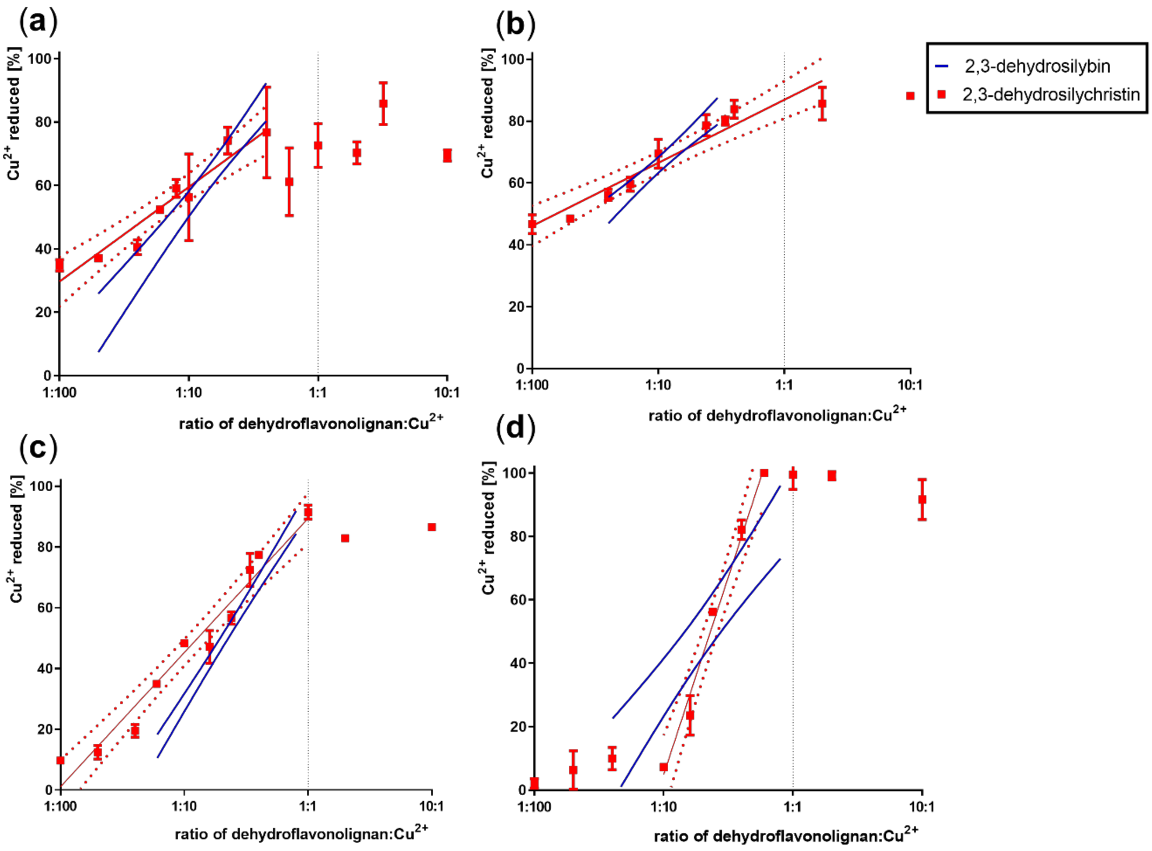

Figure S6. Comparison of cupric reduction between 2,3-dehydrosilychristin and 2,3-dehydrosilybin using 95% confidence intervals of linear regression lines.

(**a**): pH 7.5, (**b**): pH 6.8, (**c**): pH 5.5 and (**d**): pH 4.5. Data depict reduction results of measurements after 5 minutes. Data for dehydrosilybin were taken from the article Tvrdý et al., 2018 – only confidence intervals are shown for better comprehensibility.

References

Tvrdý, V.; Catapano, M.C.; Rawlik, T.; Karlíčková, J.; Biedermann, D.; Křen, V.; Mladěnka, P.; Valentová, K. Interaction of isolated silymarin flavonolignans with iron and copper. *J. Inorg. Biochem* **2018**, *189*, 115-123, doi:10.1016/j.jinorgbio.2018.09.006.
